# Association between plasma homocysteine concentration and the risk of all-cause death in adults with diastolic dysfunction in a community: A 13-year cohort study

**DOI:** 10.1097/MD.0000000000006716

**Published:** 2017-04-28

**Authors:** Jing-Ling Luo, Kuo-Liong Chien, Hsiung-Ching Hsu, Ta-Chen Su, Hung-Ju Lin, Pei-Chun Chen, Ming-Fong Chen, Yuan-Teh Lee

**Affiliations:** a Division of Cardiology, Department of Internal Medicine, Min-sheng Hospital, Taoyuan county; b Institute of Epidemiology and Preventive Medicine, College of Public School, National Taiwan University; c Department of Internal Medicine, National Taiwan University Hospital, Taipei, Taiwan.

**Keywords:** diastolic dysfunction, homocysteine, mortality, The Chin-Shan Community Cardiovascular Cohort

## Abstract

Hyperhomocysteinemia (HHCYS) has been associated with systolic heart failure. However, it is still unknown that serum homocycsteine level was useful in predicting the outcome in patients with diastolic dysfunction. We conducted a cohort study to determine if HHCYS was associated with poor prognosis in diastolic dysfunction patients. The Chin-Shan Community Cardiovascular Cohort (CCCC) study was designated to investigate the trends of cardiovascular morbidity and mortality in a community. Individuals who were 35 years and above were enrolled. Participants were categorized by homocysteine concentration quartiles. We used multivariate Cox proportional hazards models to calculate the hazard ratio (HR) of the 4th quartiles versus the 1st quartile. Area under the receiver-operating characteristic (ROC) curve was to compare prediction measures. A total of 2020 participants had completed the echocardiography examination, and 231 individuals were diagnosed as diastolic dysfunction. A total 75 participants had died during follow-up period. HHCYS was found to be significantly associated with poor prognosis. The adjusted HR for homocysteine level was 1.07 (95% confidence interval [CI], 1.01–1.14). Participants in the highest quartile had a 1.90 (95% CI, 0.88–4.12, *P* for trend, .026) fold risk for all cause death, compared with those in the lowest quartiles. The HR was 1.88 (95% CI, 1.07–3.29) using 11.11 μmol/L as cut point for hyperhomocysteine. HHCYS was significantly associated with poor prognosis in diastolic dysfunction participants in the community.

## 1 Introduction

Increased plasma homocysteine (HCY) level is associated with arterial ischemic events such as acute myocardial infarction, peripheral vascular disease, and stroke.^[1]^ Hyperhomocysteinemia (HHCYS) is also a significant marker related to cardiovascular events and all-cause death.^[2]^ Experimental and clinical data had demonstrated this relationship by showing that HHCYS in patients can lead to the prognosis of heart failure.^[3]^ However, the cellular mechanisms regarding the effects of HHCYS on cardiac remodeling and pump function are not very well understood.^[4–6]^ Previous studies had shown that the patients with HHCYS have a higher risk of left ventricular hypertrophy (LVH) and coronary artery disease (CAD).^[7–9]^ Since HCY is a potential proinflammatory and prooxidative compound. The increased level of homocycteine in the body, caused by HHCYS, may contribute to the pathogenesis of cardiovascular structures and endothelial dysfunctions.^[4,10]^ Experimental studies had shown that HHCYS may adversely affect the myocardium, leading to hypertrophy of ventricles, and a disproportionate increase in collagen.^[11–15]^ These remodeling and dysfunction can ultimately lead to the prognosis of LVH, CAD, and impaired left ventricular systolic or diastolic functions.^[16]^ However, very few studies had investigated the relationship between HHCYS and impaired left ventricular diastolic dysfunction.^[4]^ Some studies had indicated that HHCYS is associated with an increased risk of mortality in patients with systolic heart failure. However, there is no study that had demonstrated the relationship among mortality rate, HHCYS, and diastolic heart failure. Furthermore, there is no agreement among the literatures on the diagnostic cutpoint for HHCYS.^[2]^ Therefore, in this study, we prospectively investigated the association of plasma HCY with the risk of all-cause death in patients with diastolic dysfunction.

## 2 Materials and methods

### 2.1 Study design and population

The participants were enrolled in the Chin-Shan Community Cardiovascular Cohort (CCCC) Study, a prospective community-based cohort study for risks factors and outcomes of cardiovascular disease since 1990.^[2,17–22]^ The CCCC study had recruited 3502 adults from northern Taiwan, homogenous in Chinese ethnicity, and are the age of 35 years and above. The details of the CCCC study had been described in previous literatures.^[2,17,23,24]^ The study was performed in accordance with the Declaration of Helsinki and was approved by the institutional review board of the National Taiwan University Hospital, and all subjects provided their written informed consent prior to participation in the study. The following is a brief summary of the initial study: The study started in 1990 with initial cohort 3602 participants. Baseline demographic data were collected through questionnaires at enrollment. Physical examinations including measurements of weight, height, blood pressure, and electrocardiography were conducted by senior medical students. Fasting serum samples of participants were collected for biochemical assays. The research team conducted biennial prospective follow-up household visits to account for the major cardiovascular morbidity and mortality. This study was approved by the institutional review board of National Taiwan University Hospital.

### 2.2 Selection of participants

In the 1992 to 1993 follow-up period, we invited the participants to undergo echocardiographic examination for the 1st time. And a 2nd session of echocardiography examination was conducted during 1994 to 1995. The velocities of mitral inflow were measured during the 2nd session of echocardiographic examination. Among the 3602 selected participants, 2214 of which had completed echocardiographic examination. A total of 147 participants were excluded due to the absence of HCY data. Another 47 participants were excluded due to the absence of mitral inflow data. Therefore, the final study population consists of 2020 participants.

### 2.3 Measurements

Two-dimensional-guided M-mode echocardiography was performed by cardiologists according to the recommendations presented by the American Society of Echocardiography.^[25]^ Peak velocity of early (E) and late atrial (A) mitral flow were obtained from an apical 4-chamber view, by pulse wave Doppler measurements. Diastolic dysfunction was defined as a mitral inflow of E/A ratio <1, deceleration time >220 cm/s, and without impairment of systolic function.^[26–28]^ Systolic dysfunction was defined as LV ejection fraction below 40%. Body mass index and body surface area were estimated by weight and height information obtained from the period of 1994 to 1995. Left ventricular ejection fraction and left ventricular mass were calculated by means of previously established method.^[29,30]^ The left ventricular mass was further divided by the body surface area to obtain a left ventricular mass index.^[23,31]^

### 2.4 Measurements of serum HCY and other biochemical markers

The procedures of blood sampling have been reported elsewhere.^[32–34]^ All venous blood samples were drawn after a 12-hour overnight fast. These samples were refrigerated immediately and transported within 6 hours, to the National Taiwan University Hospital. Serum HCY samples were collected into tubes containing ethylene-diamine-tetra-acetic acid. The serum samples were then stored at −70 °C until analysis. HCY levels were measured by fluorescence polarizaion immunoassay (FPI; Abbott Imx System). The data from the immunoassay correlated very well with results obtained by high-performance liquid chromatography (HPLC).^[35–37]^

### 2.5 Outcome measures

The end points of this investigation were all-cause death in the follow-up period from 1994 to 2007. Deaths from any cause were identified from the official certified documents and further verified by house-to-house visits.

### 2.6 Statistical analysis

The participants in this study were categorized into quartiles by their serum HCY concentration. Continuous variables are presented as mean (SD) or median values. The categorized data are presented in the form of contingency tables. Analysis of variance (ANOVA) and chi-square tests were used to analyze the corrections between quartiles. The age and gender-adjusted Spearman partial correlation coefficients were calculated between baseline HCY concentrations and blood pressures, left ventricle mass index, lipid profiles, and fasting glucose. Incidence rates for all-cause death were calculated for each HCY quartile by dividing the number of cases by the numbers of person-years of follow-up. The hazard ratio (HR) and 95% confidence interval (CI) were determined by the multivariate Cox proportional hazards models. Logistic regression analysis was performed to determine the significance between all-cause death, and crude HCY and 4 quartiles of HCY. Three specific models were used in estimating the HRs of events, in the higher HCY quartiles relative to the lowest quartiles. In model 1, the univariate HR of HCY was estimated with the 1st quartile as the reference. In model 2, the HCY was adjusted according to the age and gender variables. In model 3, we used variables chosen by model selection. The model selection was to select the adequate variables with an entry level of 0.3, and a stay level of 0.15. The HR of these 3 models was calculated by using HCY as 4 quartiles and as an independent variable. Furthermore, a receiver-operating character (ROC) curve was constructed to generate the optimal cutoff point with highest Youden index for all-cause death. The HRs were then calculated using the resulted cutoff point.

Exist modifying factors (confounding factors) in the HCY mechanism were investigated. The patients were stratified according to the modifying factors. And the HR was calculated within each stratified group. In addition, we also introduced interaction terms into our models to test whether if these terms are the modifying factors. Each factor would be considered as a significant confounding factor the resulting logistic regression *P* value is <.05. The possible confounding factors in our models are age, gender, hypertension, diabetes, and cigarette-smoking history.

All statistical tests were performed as 2-tailed tests. Type I error of 0.05 and *P*-values <.05 were considered statistically significant. Analyses were performed with SAS software (version 9.1; SAS Institute, Cary, NC).

## 3 Results

Baseline characteristics of the participants are shown in Table 1. Participants in the highest quartile were less likely to be female. They were likely to smoke cigarettes, drink alcohol, and had a higher prevalence of CAD history. Furthermore, the highest quartiles were older, had higher blood pressure, and had higher left ventricular mass index.

**Table 1:**
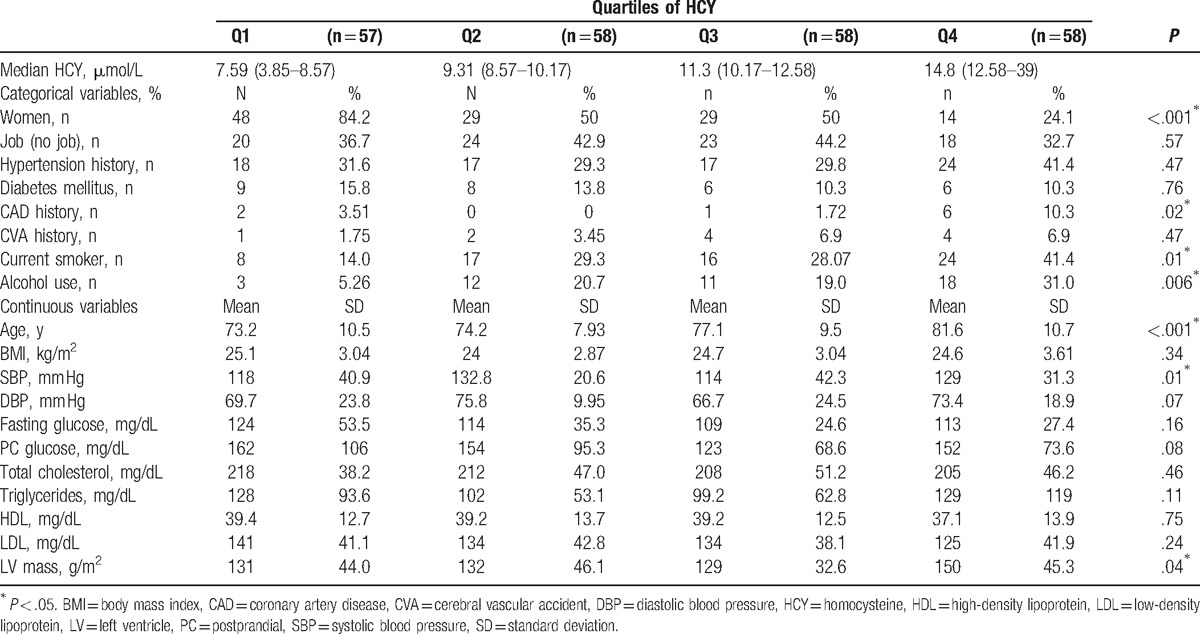
Characteristics of the study population according to HCY quartiles.

Among the 3602 selected participants, 2020 participants constituted this study population. In these 2020 participants, 231 adults had diastolic dysfunction, which was defined as a mitral inflow E/A ratio of <1, deceleration time of >220 cm/s, and without systolic dysfunction.

The average HCY level among the 231 adults was 11.1 μmol/L, the inter quartile ranged between 8.5 and 12.6 μmol/L. The relationship between HCY level and other variables was investigated by gender-adjusted Spearman partial correlation coefficients. Our study had shown that there were no statistically significant correlations between HCY concentrations and blood pressure, left ventricle mass index, lipid profiles, and fasting glucose.

**Figure 1:**
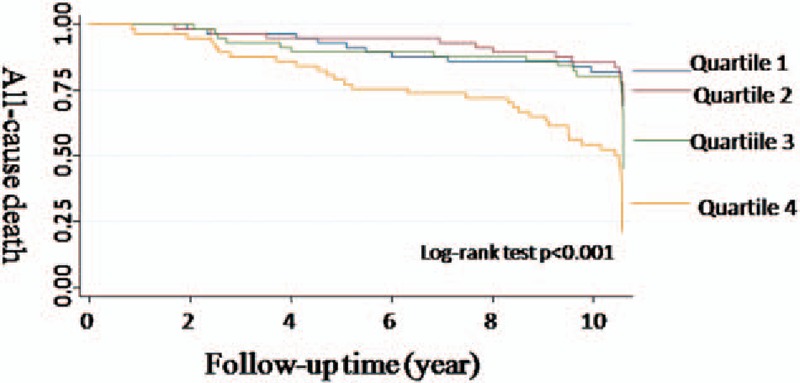
Kaplan–Maier curves of the cumulative probability of death according to quartiles of homocysteine (HCY). According to Kaplan–Meier analysis, there is significant difference between HCY quartiles. HCY concentration is strongly associated with the risk of death among adults, of Chinese ethnicity, with diastolic dysfunction. Log rank test *P* < .001 for Q4 versus Q1.

Among the 231 participants, the median follow-up person-year was 10.5 years with interquartile range between 9.52 and 10.6 person-year. There were 75 cases of deaths documented. The incidence rates of all-cause death increased with HCY quartiles (Fig. 1). The incidence rate of all-caused death in the highest quartiles and lowest quartiles were 73.8 and 23.9 per thousand person-year, respectively (Table 2). Finally, the variables such as age, gender, cigarette-smoking history, cholesterol, fasting glucose, and hypertension history were used in model 3. After adjusting the risks factors (model 3), the HR for participants in the highest quartile of HCY compared with those in the lowest quartile were 1.90 (95% CI 0.88–4.12, *P* for trend, .026) (Table 3). The HRs were then verified, using HCY as continuous variables. The HR of model 3 was 1.07 (95% CI 1.01–1.14, *P* = .016) (Table 4).

**Table 2:**
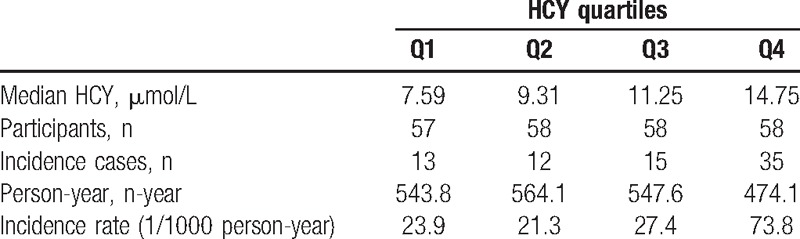
Median homocysteine (HCY) concentration, numbers of participants, incidence cases, person-year, and incidence rate by HCY quartiles.

**Table 3:**
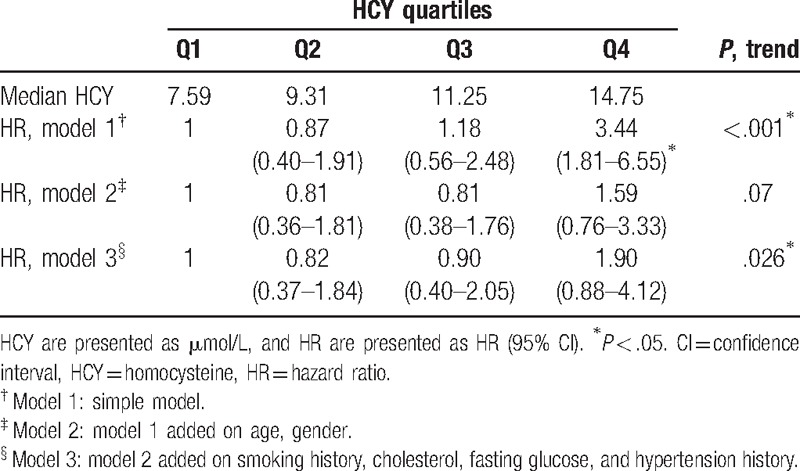
HRs and 95% CI by HCY quartiles for the association of all-cause death.

**Table 4:**
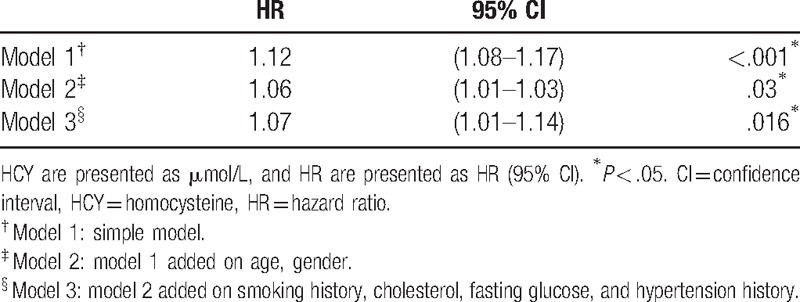
HRs and 95% CI by HCY in continuous variable for the association of all-cause death.

In our study, an ROC curve was constructed to determine the optimal cutpoint of HCY in predicting the all-cause death. The optimal cutpoint with highest Youden index (sum of sensitivity and specificity-1) was 11.11 μmol/L. The sensitivity was 64%, specificity was 71.2%, and the area under curve was 0.68 at this cutpoint. We then used this cutpoint of HCY to calculate the HR for the participants in the higher HCY quartile. The HR for these participants was determined to be 1.88 (95% CI 1.07–3.29, *P* = .028) (Table 5).

**Table 5:**
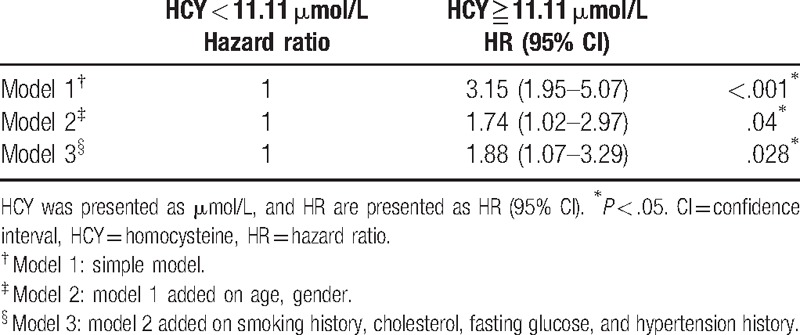
HR for all-cause death using 11.11 μmol/L as cutpoint.

In our study, we also investigated the other possible confounding factors such as age, gender, hypertension, diabetes, and cigarette smoking history. We had used 75-year old as a cut point for age. We found that in participants who were older the 75 years old, and belonged to the 4th quartile, had a higher mortality rate compared to that of the 1st quartile. The HR was 2.68 (95% CI, 1.09–6.56, *P* < .001, *P* for interaction .29) for these participants. Also, in participants older than 75 years old, the HR of HCY was 1.12 (95% CI, 1.06–1.17). And in participants younger the 75 years old, the HR was 0.96 (95% CI, 0.79–1.17). With the aforementioned results, we can conclude that age is an effect modifier. Other factors such as gender, hypertension, diabetes, and cigarette-smoking history were also studied in this analysis. These factors, including gender, hypertension, diabetes, and cigarette smoking history, did not modify the mechanism, and therefore are not effective modifiers (Table 6).

**Table 6:**
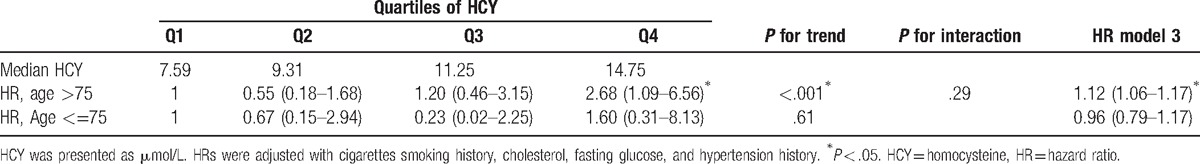
Age-stratified HR, *P* for trend, and *P* for interaction in the study participants.

## 4 Discussion

In this study, we demonstrated that serum HCY concentration is strongly associated with the risk of death among adults, of Chinese ethnicity, with diastolic dysfunction. We also had found that the optimal cutpoints for HCY concentration in this population by using Youden index. Furthermore, age was determined to be important factors in the mechanism which homocycsteine participates in. HCY was shown to be a good predictor for all-cause death among old Chinese adults. Several evidences had shown that HCY is associated with poor prognosis in cardiovascular disease patients. This study is the first cohort study to evaluate the relationship between HCY and all-cause death in patients with diastolic dysfunction.

Several pathogenic mechanisms had shown that HCY was associated with diastolic dysfunction, including endothelial dysfunction and smooth muscle proliferation.^[11]^ However, oxidative stress, activation of protein kinase C, and altered collagen metabolism had also played important roles in the mechanism.^[38–40]^ Furthermore, HHCYS may increase cardiac fibrosis and the activation of matrix metalloproteinases, where in turn can lead to the remodeling of the left ventricle.^[3]^ HCY was shown to promote smooth muscle proliferation, which can lead to LVH.^[29]^ LVH is strongly associated with diastolic dysfunctions. A previous cohort study had showed that plasma HCY is directly related to left ventricular mass and wall thickness.^[29]^ These result had shown a strong relationship between plasma HCY levels to cardiovascular disease and diastolic dysfunction.^[29]^

Previous studies had showed that HHCYS is associated with poor prognosis in patients with congestive heart failure. The cutpoint of homocycteine concentration and HR were determined to be 14 μmol/L and 3.26 (95% CI, 1.78–5.98), respectively.^[41]^ Another study had indicated that the all-cause mortality was strongly associated with the level of plasma HCY. The cutpoint of homocycteine concentration and HR were determined to be 11.84 μmol/L as cutpoint and 2.4 (95% CI, 1.76–3.32), respectively.^[2]^ In our study, the adequate homocycteine concentration cutpoint was 11.11 μmol/L. The HR was 1.88 (95%CI, 1.07–3.29). The variation among the reported values is due to the fact that cutpoint and the HR were highly dependent on the study population. In general, if the study population had a higher disease severity, the HR of this population would also be higher. Our study population consisted with patients with diastolic dysfunction were relatively healthier than the patients with congestive heart failure. So the HR value, in patients with diastolic dysfunctions, was lower than the that of the HR value of patients with congestive heart failures.^[41]^

In this study, we also tested age, gender, hypertension, diabetes, and smoking history as the possible hypermohocysteinemia confounding factors. We had found that patient age is a possible effecting modifier. In participants older than 75 years old, HCY significantly increased with mortality rate. The HR for these older participants was 1.12 (95% CI, 1.06–1.17). However, this relationship was not observed in participants younger the 75 years old. Therefore, we suggest that the importance of HCY increases proportionally with the age of the patient. This is especially true when using homocycteine levels as a mortality predictor in older patients. This result is in concurrence with a previous study, where the concentration of HCY can become a cardiovascular mortality predator in patients of very old age.^[42]^ This study also suggests that the concentration of HCY is a better predictor then the classic risk factors for patients of very old age.

### 4.1 Strengths and weaknesses

Our study has several strengths. First of all, this is the first cohort study of HCY and all-cause mortality in diastolic dysfunction participants. HCY was shown to be an important marker for diastolic dysfunction patients of very old age. We also constructed an ROC curve to determine the optimal cutpoint of homocystine level in diastolic dysfunction participants. This study enrolled 231 diastolic dysfunction participants, and follow-up was performed for up to 13 years.

Our study also had some limitations. First of all, we lacked information on some determinants of total HCY level such as dietary patterns, folic acid, and fortification of food and vitamin supplements. Second, we determined diastolic dysfunction only by mitral inflow, which may not be sufficient, and the number of participants with diastolic dysfunction may be underestimated. Finally, in this study we had used the all-caused death for outcome management due to the fact that we lacked a complete clinical information such as cardiovascular events, etc.

## 5 Conclusion

In this cohort study, we had shown that people with diastolic dysfunction and a higher level of HCY have a significant higher risk of all-cause death. Plasma HCY level was a good predictor for all-cause death among old adults.
